# Invariant Natural Killer T Cell Subsets—More Than Just Developmental Intermediates

**DOI:** 10.3389/fimmu.2018.01393

**Published:** 2018-06-20

**Authors:** S. Harsha Krovi, Laurent Gapin

**Affiliations:** ^1^Department of Immunology and Microbiology, University of Colorado Anschutz Medical Campus, Aurora, CO, United States; ^2^Department of Biomedical Research, National Jewish Health, Denver, CO, United States

**Keywords:** invariant natural killer T cells, subsets, development, homeostasis, cytokine secretion

## Abstract

Invariant natural killer T (iNKT) cells are a CD1d-restricted T cell population that can respond to lipid antigenic stimulation within minutes by secreting a wide variety of cytokines. This broad functional scope has placed iNKT cells at the frontlines of many kinds of immune responses. Although the diverse functional capacities of iNKT cells have long been acknowledged, only recently have distinct iNKT cell subsets, each with a marked functional predisposition, been appreciated. Furthermore, the subsets can frequently occupy distinct niches in different tissues and sometimes establish long-term tissue residency where they can impact homeostasis and respond quickly when they sense perturbations. In this review, we discuss the developmental origins of the iNKT cell subsets, their localization patterns, and detail what is known about how different subsets specifically influence their surroundings in conditions of steady and diseased states.

## Introduction

Adaptive immunity has long been appreciated as a chief means through which various jawed vertebrates stave off infectious pathogens. One of the main features differentiating the adaptive immune system from its innate counterpart is the generation and expression of diverse antigen receptors. Antigen sensors of the innate immune system are proteins with fixed sequences and pattern-recognition motifs encoded within the germline (1). By contrast, antigen receptor generation by adaptive immune cells involves complex somatic DNA rearrangements that juxtapose genes otherwise separated by thousands to millions of base pairs (2). Each cell randomly rearranges its antigen receptor locus to ensure that few cells express identical receptors. In so doing, the cells express clonal receptors and develop an antigen receptor repertoire diverse enough and with the potential to recognize the plethora of existing antigens that the host is likely to encounter. Because the antigen receptors in the adaptive immune system are heterodimers, with both subunits undergoing independent rearrangements, the combinatorial diversity has been estimated to exceed 10^15^ unique sequences (3).

The two types of cells belonging to the adaptive immune system, B and T lymphocytes, have each evolved in different ways to efficiently respond to infections. In particular, B cells produce and secrete antibodies that target and bind to different conformational epitopes on pathogens (4). T cells, on the other hand, express cell membrane-tethered antigen receptors (TCRs, composed of α-chains paired to β-chains), thereby necessitating their proximity to their targets in order to initiate an immune response. These TCRs primarily recognize their target ligands by interacting with major histocompatibility complex (MHC) proteins, which present linearized peptides, expressed on adjacent cells (5). The presented peptides are processed fragments from full length proteins and can be presented by MHC-I molecules interacting with TCRs expressed on CD8^+^ T cells or MHC-II proteins interacting with TCRs expressed on CD4^+^ T cells (6). Correspondingly, these T cells not only interact with different MHC molecules but also produce distinct responses.

To ensure that T cells are capable of mounting immune responses against all kinds of invading pathogens, T cells have further evolved to differentiate into functionally distinct subsets. Indeed, CD4^+^ T cells can differentiate into T_H_1, T_H_2, T_H_17, T_reg_, among others, upon exposure to their cognate antigens (7). Each of the subsets produces a distinct set of cytokines with the capacity to skew the immune response in a specific direction. For example, T_H_1 cells produce IFNγ, a pro-inflammatory cytokine that promotes increased antigen presentation by MHC molecules and increased phagocytosis by macrophages, to name a few of its effects. CD4^+^ T cell differentiation into T_H_1 cells is thought to be primarily due to intracellular pathogens (8). T_H_2 cells, though, produce a different set of cytokines, including IL-4, IL-5, and IL-13, and assist in combating extracellular pathogens such as parasites and helminths (9). Through this division of labor, functionally different T cell subsets can resolve infections by producing responses catered to the pathogen.

The substantial TCR repertoire diversity in the adaptive immune system serves as a double-edged sword. Many different TCRs are expressed by the T cell population *en masse*, but only a few T cells expressing TCRs specific for a given antigen exist within the population (10). Thus, T cells undergo extensive proliferation when they first encounter their antigen to generate enough cells with the proper antigen-specific TCRs. The activated cells can then travel to the site of infection and execute their appropriate functions upon antigen re-challenge. As this process usually takes several days, the adaptive immune system is considered a slow and deliberate yet specific form of response. After the immune response has been resolved, some of the cells differentiate into memory cells, which exhibit faster response times in the event that the pathogen reinfects the host (11).

Due to the delayed kinetics of this “conventional” arm of the adaptive immune system, other “innate-like” adaptive lymphocytes play a crucial role early during an infection (12). These cells are unique because they express markers associated with memory cells despite not having encountered their antigens previously. Additionally, they are functionally poised and capable of responding within hours as opposed to days, in line with their innate-like capabilities. Though they make up only a fraction of the overall T cell population, they still exert crucial and sometimes non-redundant functions. One such lymphocyte population, which will be the focus of this review, is the invariant natural killer T (iNKT) cell lineage. These cells straddle the innate-adaptive boundary because they respond quickly upon stimulation (within hours), yet, express a TCR that underwent somatic rearrangement (13). Indeed, the vast majority of iNKT cells express an identical TCRα chain (TRAV11-TRAJ18 in mice, TRAV10-TRAJ18 in humans) paired to a restricted set of TCRβ chains (TRBV1, TRBV13, and TRBV29 in mice, TRBV25 in humans), with some notable exceptions (14–16). Furthermore, instead of interacting with peptides presented by MHC molecules, the TCRs expressed by iNKT cells recognize (glyco)lipids presented by CD1d, a non-polymorphic MHC-I-like molecule (17).

Analogous to the aforementioned functionally different conventional T cell subsets, iNKT cells also come in different flavors, each of which exhibits a different functional profile (18–20). Such division of labor between functionally different iNKT cell subsets perhaps could explain why iNKT cells have been implicated in ameliorating or exacerbating a variety of diseases and illnesses ranging from autoimmunity to cancer. Historically, iNKT cells have been lumped into one category despite their varied roles in responses. However, with the recent identification of functionally distinct iNKT cell subsets, how and which iNKT cell subsets might affect the development of the immune system and its response need to be updated. In this review, we will focus on how the different iNKT cell subsets develop and consequently, to what extent each of these subsets actively participates in immune responses.

## iNKT Subsets

Initially, an intriguing population of mature T cells was identified in the thymus by their lack of expression of CD4 or CD8 coreceptors (double negative, DN) but with surface expression of a TCR, thereby distinguishing them from other immature thymocytes (21). These DN cells were functionally competent since they could produce IL-4 and IFNγ readily after stimulation and also expressed the natural killer (NK) cell marker NK1.1 (22–24). This was particularly novel because T cells were not traditionally considered to be cytokine secretion-competent in the thymus and suggested that functional competence by these cells might be acquired during their development. Sequencing the TCRs from these cells repeatedly provided investigators with the same TCRα chain sequence (25, 26), and it was eventually determined that the cells required CD1d expression for their development, suggesting that they recognized lipids instead of peptides (27). Due to the expression of a TCR as well as NK markers by these cells, the name iNKT took preferential hold as a label for these cells.

With the discovery of the marine sponge-derived lipid α-galactosylceramide (αGC) that when bound to CD1d strongly stimulated these cells and the advent of MHC-loaded tetramer technology, iNKT cells could now be tracked with profound resolution (28–30). Interestingly, it became readily apparent that not all the cells that were identified by αGC-loaded CD1d tetramers were NK1.1^+^, suggesting phenotypic heterogeneity within the iNKT compartment. Because the NK1.1^+^ cells composed the overwhelming majority of the total tetramer^+^ population in the thymus in C57BL/6 (B6) mice, the NK1.1^−^ cells were thought to perhaps represent developmental intermediates. Indeed, support for this idea came from experiments in which intrathymic transfers of NK1.1^−^ cells could generate NK1.1^+^ cells (31, 32). Interestingly, stimulating the NK1.1^−^ cells led to the production of larger amounts of IL-4 compared to IFNγ, in stark contrast to what the NK1.1^+^ cells produced, which was primarily IFNγ and little IL-4 (31–33). Additionally, the iNKT cells that were primarily exported from the thymus were NK1.1^−^ cells while the NK1.1^+^ cells were retained in the thymus (34, 35). Thus, it was unclear why the intermediates had a different cytokine secretion profile compared to the terminally matured population and furthermore, why/how the immature cells emigrated from the thymus if they were truly meant to give rise to the mature iNKT cells (36).

Only recently has this conundrum been resolved due in large part to the work by the Hogquist group. Instead of identifying iNKT cells simply by the tetramer and NK1.1, they also stained the cells with transcription factors known to endow specific fates (19). Because of years of work in understanding conventional CD4^+^ T cell differentiation, it was known that the master transcription factors engendering the T_H_1, T_H_2, and T_H_17 fates were T-bet, GATA-3, and RORγt, respectively (8). In addition, large scale screens by two groups had recently identified that all iNKT cells require the expression of the zinc finger transcription factor promyelotic leukemia zinc finger (PLZF) (37, 38). In fact, the few remaining iNKT cells (as identified by the tetramer) found in PLZF-deficient mice resembled naïve T cells that secreted IL-2 upon stimulation but not significant levels of IL-4 or IFNγ, highlighting the transcription factor’s importance (37, 38). By using antibodies targeting T-bet, GATA-3, RORγt, and PLZF, Hogquist and colleagues had the surprising finding that not all thymic iNKT cells expressed each of the transcription factors. Instead, three distinct thymic subpopulations were identified based on their staining: PLZF^hi^ GATA-3^hi^ (iNKT2), PLZF^int^ RORγt^+^ (iNKT17), and PLZF^lo^, T-bet^+^ (iNKT1) (19). Moreover, NK1.1 primarily stained the iNKT1 cells. Thus, although a few iNKT1 precursors were present in this pool, many of the NK1.1^−^ cells were terminally differentiated cells themselves. This was further confirmed by stimulating the different subsets *in vitro*, with iNKT1 cells producing large amounts of IFNγ and a little IL-4, iNKT2 cells producing large amounts of IL-4, and iNKT17 cells secreting IL-17, putting to rest the functional discrepancy between NK1.1^+^ and NK1.1^−^ cells (19).

Interestingly, not all thymic iNKT subsets are equally represented in different strains of mice. Certain strains, such as the BALB/c, have large proportions of iNKT2 and iNKT17 cells with a correspondingly reduced proportion of iNKT1 cells. On the other extreme, B6 mice instead possess largely iNKT1 cells and few iNKT2/iNKT17 cells (19). This is particularly important because previous work in these mice had led to the prevalent idea that, generally speaking, B6 mice tended to be predisposed to a “T_H_1 phenotype” while BALB/c mice displayed a “T_H_2 phenotype” (39, 40). Whether the iNKT cell compositions in either of these strains are a cause or a consequence of these phenotypes is unknown. Another point of note is that the reason for the overrepresentation of NK1.1^+^ iNKT cells in previous experiments was the predominant use of the B6 mouse for the study of iNKT cell development. In fact, the antibody targeting NK1.1 in B6 mice does not recognize the epitope present in BALB/c mice due to an allelic variance (41). The use of this antibody necessitated experiments in B6 mice since the cells were initially characterized by their expression of NK1.1 (22). Now, however, iNKT cells in all strains are identified by their ability to interact with the αGC (or its analog PBS57) loaded CD1d tetramer and their transcription factor profile (or surface proteins known to be specifically upregulated by these transcription factors) serves as a readout of the subset proportions.

The terminal differentiation status of these subsets has also been challenged due to the discovery of new iNKT cell subsets in the periphery. Although only the three aforementioned subsets are largely represented in the thymus, analysis of other tissues in both steady-state and immunization conditions has revealed the presence of novel iNKT subsets. In the adipose tissue, a special iNKT cell population, named iNKT10, has been identified that depends on expression of the transcription factor E4BP4 for its role in maintaining adipose tissue homeostasis (42). Similarly, an iNKT_FH_ population expressing the transcription factor Bcl6 has been observed in the peripheral lymphoid organs of immunized mice (43). This population secreted IL-21 and provided cognate help for B cells undergoing affinity maturation, much like conventional T_FH_ cells in germinal centers (43–45). Ongoing work should help determine whether these additional subsets are indeed generated at low frequencies in the thymus or if they differentiate into their observed subsets within other tissues.

## iNKT Cell Subset Development

**Figure 1:**
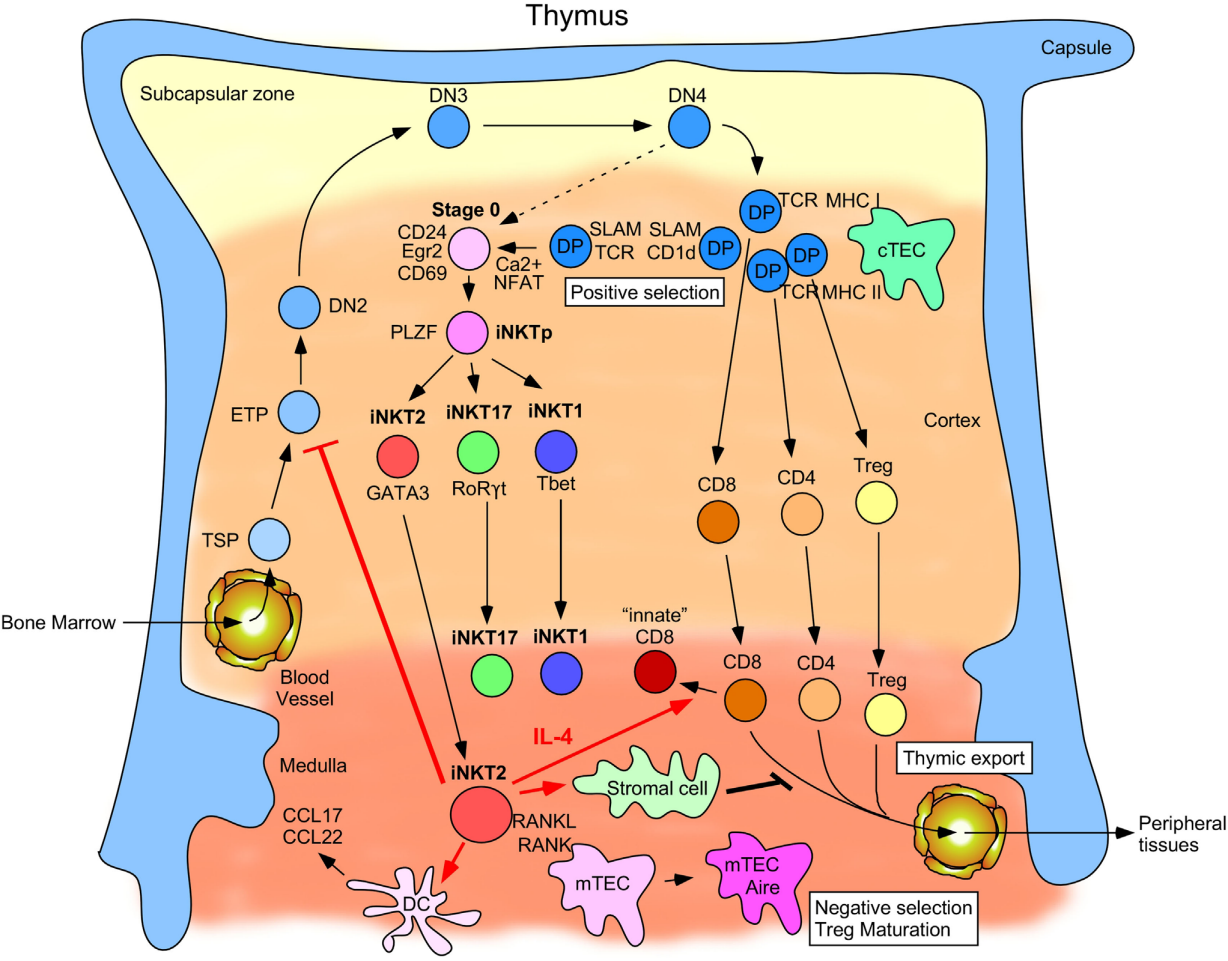
Schematic describing invariant natural killer T (iNKT) cell development and function in the thymus. Thymus-settling progenitors emigrate from the bone marrow and then mature into early thymic progenitors and progressively commit to the T cell lineage by maturing from the double negative 2 cell stage to DN3, DN4, and eventually to the double-positive (DP) cell stage. Here, they begin to rearrange their TCRα loci and can then be selected by MHC-I- and MHC-II-expressing cortical thymic epithelial cells (cTECs). Cells that successfully undergo positive selection by cTECs can become immature CD8^+^, CD4^+^, and T_reg_ cells. By contrast, a small proportion of DP cells rearranges its TCRα locus to generate the iNKTα chain and is selected by other DP cells expressing CD1d and signaling lymphocytic activated molecules family receptors. Intracellular calcium levels are high in these post-selected stage 0 cells, which leads to NFAT translocation into the nucleus and upregulation of Egr2 and CD69. An alternative pathway (depicted by a dashed arrow) wherein a small number of DN4 thymocytes rearrange their TCRα loci to generate the iNKTα chain that can then give rise to stage 0 iNKT cells upon positive selection has also been described. These cells then transition through an uncommitted PLZF^hi^ stage before diverging into the functionally distinct iNKT2, iNKT17, and iNKT1 subsets defined by the transcription factors GATA-3, RORγt, and T-bet, respectively. iNKT2 cells migrate from the cortex to the medulla where they begin to produce IL-4 at steady-state. This IL-4 production (as designated by red arrows) has been linked to conditioning surrounding CD8^+^ T cells to become innate-memory CD8^+^ T cells, promoting certain dendritic cell populations to secrete the chemokines CCL17 and CCL22, preventing ETP commitment to the T cell lineage and inhibiting thymic export of single positive (SP) thymocytes into peripheral tissues. RANKL expressed by medullary iNKT2 (and iNKT17) cells also promotes maturation of medullary thymic epithelial cells (mTECs) into Aire^+^ MHC-II^hi^ mTECs, which mediate negative selection of medullary SP thymocytes and T_reg_ maturation.

CD4^+^ CD8^+^ [double positive (DP)] thymocytes serve as the progenitors for all cells belonging to the αβ T cell lineage (46, 47). iNKT cells are no different as they also principally originate from DP precursors (48, 49), with a minor proportion utilizing an alternative pathway (Figure 1) (50). DP cells randomly rearrange their TCRα loci to generate the invariant TCRα chains that pair with suitable TCRβ chains (49). While DP precursors of conventional T cells are selected by MHC I/II on thymic epithelial cells (TECs), iNKT cell DP precursors are instead positively selected by self-lipids presented by CD1d expressed on fellow DP thymocytes (51, 52). The DP–DP interaction provides the iNKT precursor with the obligate lipid/CD1d ligand along with a distinctive homotypic co-stimulation through members of the signaling lymphocytic activated molecules (SLAM) family of receptors. Signals derived from SLAM family receptor interactions are required to produce mature iNKT cells because iNKT cells are notably absent in mice in which an adapter downstream of SLAM receptors (SAP) has been deleted (38, 53). Interestingly, different mouse strains also express different alleles of the SLAM receptors. For example, BALB/c mice possess an allele of SLAMF6 (Ly108) that is hyperphosphorylated upon engagement compared to the B6 Ly108 allele (54, 55). This hyperphosphorylation has a functional effect because a stronger signal is consequently transduced in BALB/c DP thymocytes (56), suggesting that signals received by iNKT cell precursors during development in the thymus might not be equivalent across mouse strains.

Co-stimulations of DP cells *via* the TCR and the SLAM receptors elicit a strong signal in the iNKT precursors leading to a high expression of the transcription factor Egr2 (56). Without Egr2, thymocytes are arrested early during iNKT cell development (57–59). High expression of Egr2 is dispensable for conventional T cell development (57), suggesting that iNKT cells are unique in their requirement for stronger-than-normal agonistic signals to properly mature. Indeed, post-positive selection iNKT cells, commonly referred to as stage 0 iNKT cells, expressed the highest levels of Nur77 (encoded by *Nr4a1*), an immediate early protein induced upon TCR signaling, compared to all other thymocytes (60). Furthermore, Egr2 has been demonstrated to bind directly to the promoter and positively regulate the transcription of *Zbtb16*, the gene encoding PLZF. In the absence of Egr2 and its related protein Egr1, PLZF levels in the few remaining iNKT cells are significantly lower, further corroborating the link between strong TCR/SLAM signaling and proper iNKT development (59). iNKT cells are imbued with an activated/memory phenotype relatively early during their ontogeny, primarily due to their expression of PLZF (37, 38). In fact, ectopic expression of PLZF is sufficient to promote a memory phenotype even in conventional CD4^+^ T cells at steady state (38, 61).

One of the outstanding questions in iNKT cell biology currently is how iNKT cell subset differentiation occurs in the thymus. Conventional T cells in the periphery require antigen-induced priming as well as the appropriate cytokine milieu to be properly polarized into different functional subsets. For example, generating T_H_1 cells from naïve CD4^+^ T cells requires TCR-mediated signals in addition to the cytokine IL-12 that promotes commitment to the T_H_1 lineage through the actions of the transcription factor signal transducer and activator of transcription 4 (7). T_H_2 cells, though, require TCR-mediated signals in conjunction with IL-4 to be polarized into their lineage (9). Polarization into different lineages in this manner has been demonstrated to be dependent on specific factors both *in vitro* and *in vivo*. However, since iNKT cells differentiate in the thymus unlike conventional T cells that differentiate in the periphery, it is unclear if they follow a similar differentiation course.

Several factors, besides the canonical transcription factors, have been revealed to be relevant for specific iNKT cell subset development and maintenance, primarily through the use of knockout mice. The transcription factor lymphoid enhancer factor 1 (Lef-1) was shown to influence differentiation into the iNKT2 lineage (62). Lef-1 expression strongly correlates with PLZF expression and Lef-1^−/−^ mice have significantly fewer iNKT2 cells capable of producing IL-4 (62). Mice lacking the serine protease Serpinb1 generate more iNKT17 cells in the thymus and more iNKT17 cells are found in peripheral tissues in KO mice as well (63). When the transcription factor Bcl11b was specifically knocked out of PLZF^+^ cells using a PLZF-Cre mouse, iNKT17 cells were preferentially maintained at the expense of iNKT1 and iNKT2 cells, suggesting that Bcl11b is required for iNKT1 and iNKT2 cell survival but is dispensable for iNKT17 cell survival (64). The transcription factor Th-POK appears to restrict the generation of iNKT17 cells because in its absence, iNKT17 cell numbers are increased and more iNKT cells are capable of producing IL-17 (65). Th-POK itself is epigenetically regulated by the micro-RNA miR133b and more iNKT17 cells are observed in mouse strains where iNKT cells express higher levels of this miRNA (66). In this manner, many other factors have similarly been described to be preferentially important for the development and/or maintenance of one subset over others. Despite the abundance of studies reporting proteins required for different subsets, what is unknown is the stage of development during which these proteins are first expressed. In other words, it is unclear if these factors themselves are the force behind the development of specific subsets or are instead a consequence of commitment to a given subset. If the former, then, different precursors should exist, each with a specific phenotype, which predispose cells into a given subset. Support for this hypothesis is currently lacking. Instead, it has been shown that DP precursors exist in a poised state on a population level. Approximately 1,000 genes in bulk DP cells are transcriptionally silent yet possess both permissive (H3K4me3) and repressive (H3K27me3) histone modifications at their transcriptional start sites (67, 68). About 14% of these genes code for transcription factors, some of which are implicated in influencing lineage diversification. These results suggest that a given DP precursor is capable of adopting various fates but only commits to one upon receiving specific cues (68). Thus, differential signals received by DP thymocytes consequently could drive the commitment of individual precursors into distinct lineages that are then preferentially dependent on specific proteins.

Several hypotheses, not all of which are mutually exclusive, can be formulated to explain how iNKT cell subsets might arise from differential signaling during development. First, the cells might enter distinct pathways, as observed in the periphery, due to stimulation by different cytokines that impose commitment to a specific lineage. In support of this idea, each of the thymic subsets expresses a unique composition of cytokine receptors (19, 69). Indeed, iNKT1 cells display a dependence on the cytokine IL-15 for survival while iNKT17 cells instead require IL-7 to maintain their numbers (70, 71). In addition, iNKT2 and iNKT17 cells also seem to require IL-25 for homeostasis and function (72). However, this idea pre-supposes that commitment only occurs after upregulation of cytokine receptors that can enforce lineage specification. Instead, cytokine receptor expression frequently occurs as a consequence of expression of particular transcription factors themselves (73–75). Because expression of the transcription factors would already signify commitment, this implies that an even earlier event drove the cells to differentially upregulate these proteins. Furthermore, stage 0 iNKT cells do not express any appreciable levels of transcripts coding for lineage-determining transcription factors, even at the single cell level (69). Instead, their transcriptomes are reminiscent of uncommitted DP cells having recently undergone positive selection, suggesting that although commitment to a subset can be reinforced by cytokine receptor signaling, it is unlikely to be the original signal driving diversification.

Another hypothesis is that recognition of specific self-ligands during selection has the potential to shape the subset ratio. Accordingly, in a recent study, antigen-specific iNKT cells were readily identified in the thymus when using CD1d tetramers loaded with a variety of lipids (76). Even though all iNKT cells could be identified using tetramers loaded with the potent antigen αGC, different subpopulations reacted exclusively to specific other lipids. Although the authors did not further categorize the responding cells based on their functional subsets, it remains an appealing idea that differential recognition of CD1d-presented lipids might have dramatic consequences for iNKT precursors. In this scenario, lineage commitment would be expected to occur early after positive selection for each cell. Different microenvironments within the thymus could present high levels of specific lipids, thereby specifically promoting selection of certain iNKT cell subsets. This seems an unlikely proposition because selection itself has been demonstrated to occur on cortical DP thymocytes while the majority of the mature subsets (approximately 70%) take up residence in the medulla of the thymus, implying that migration to different thymic niches occurs well after positive selection (77). It is instead plausible that the precursors encounter different antigens merely by chance in a homogenous cortical environment, although this possibility remains to be formally demonstrated.

Different ligands, though, are only distinguished by iNKT DP precursors through the use of a diverse TCR repertoire (19, 78, 79), raising a third non-mutually exclusive possibility that iNKT cell subsets might arise due to differential signaling transduced by their TCR during positive selection. As the cells undergo selection, the strength of the signal perceived by each cell due to the nature of the TCR as well as the specific ligand being recognized could instruct each cell to adopt and commit to a specific lineage. This is an intriguing idea since TCR signal strength influencing fate decisions has been demonstrated in a variety of contexts (80–82). In addition, at the population level, it is reasonable to postulate that the precursor cells express an even distribution for a variety of markers, precluding the predisposition of any one cell to enter a given pathway. However, the nature of the αβ TCR (the TCRβ chain in particular in the case of iNKT cells) does vary from cell to cell, making it likely that the signals transduced by the TCRs could similarly vary. The signals thus generated might have a small range of strengths but through co-stimulation by SLAM receptors (and perhaps other coreceptors), this range could be amplified and engender distinct fates to cells that land on either end of the spectrum. In agreement with this, mature iNKT subsets express different levels of Nur77, with iNKT2 cells expressing the highest, followed by iNKT17 cells, and finally with iNKT1 cells expressing the lowest levels (19). Using Egr2 as a marker for strength of TCR-mediated signals during positive selection, data generated in our lab also confirm this hierarchy (83). Thus, the cells within the subsets seem to retain a memory of the signals they received as precursors, with iNKT2 cells having received the strongest signals followed by iNKT17 and iNKT1 cells.

One way that cells might retain the signaling information could be through the transcription factor GATA-3. TCR signaling has been previously demonstrated to upregulate GATA-3 protein levels (84). Many of the genes coding for the components of the TCR complex, namely the *Tcra, Cd3d*, and *Cd3g* loci, are direct targets of GATA-3 (75, 85–87). In mice lacking GATA-3, expression of these different genes is significantly reduced. Furthermore, GATA-3 has also been previously shown to autoregulate its own expression in a positive feedback loop (88). Therefore, stronger signaling during positive selection could potentially lead to higher and sustained GATA-3 levels and consequently, higher TCR levels. In support of this, the TCR levels (and GATA-3 levels to some extent) on the different subsets follow the same pattern as Nur77 and Egr2 do, perhaps suggesting that signals received during selection could be maintained in this manner (19, 63).

Pairing the invariant TCRα chain with different TCRβ chains can also affect the affinity with which the TCR heterodimer interacts with antigen/CD1d and consequently, how efficiently the TCR can initiate and propagate a signal intracellularly (89). Interestingly, in retrogenic mice generated with distinct TCRβ chains, the proportions of each of the subsets could be linked to the avidity of the TCR for its ligand (90). Similarly, when clonal mice were generated using nuclei from iNKT cells expressing different TCRs, the proportion of PLZF^hi^ iNKT cells in the thymus directly correlated with the avidity of the TCR for lipid/CD1d (91). Finally, different studies have revealed that TCR signaling regulates the expression levels of several proteins involved in chromatin remodeling and in whose absence, the subset ratios are vastly altered (68, 92, 93). With the advent of myriad technologies allowing immunologists to assess transcriptomic and epigenomic signatures at the resolution of a single cell, it will become paramount in the future to pursue single cell analyses on the stage 0 iNKT cells immediately following positive selection and determine if TCR signaling-mediated differences can already be identified within these cells. Although a recent study did conduct single-cell RNA-sequencing analysis on stage 0 iNKT cells, the study concluded that these cells were similar to other positively selected conventional cells (69). As this study only analyzed 45 stage 0 iNKT cells, obtaining greater depth by sequencing more stage 0 iNKT cells could potentially provide more information on otherwise non-sampled low-abundance transcripts and/or accessible loci in different cells. With this information, perhaps an early signature can be identified that correlates with eventual iNKT cell subset.

## iNKT Subset Tissue Homeostasis

After developing in the thymus, iNKT cells have been observed in various tissues throughout the body (13). Unfortunately, due to an incomplete understanding of iNKT cell subsets, only their presence or absence in various tissues could be ascertained until recently. Some studies had identified iNKT cells in different tissues by αGC-CD1d tetramer staining, which remains the gold standard (30, 94, 95). This staining, however, was rarely done in conjunction with staining for the master transcription factors associated with the subsets, precluding their identification. In other studies, cells were frequently identified by their co-expression of NK1.1 and TCRβ (78, 96, 97). This strategy is perhaps problematic for multiple reasons. First, since staining for NK1.1 is not successful in all strains (41), it is entirely possible that observations made using the B6 mouse model are not generalizable to all mouse strains, as demonstrated in BALB/c and non-obese diabetic (NOD) NK1.1-congenic mice (98). Second, NK1.1 does not exclusively mark iNKT cells as conventional CD8^+^ T cells can also co-express NK1.1, potentially obfuscating the real iNKT population (99, 100). Indeed, cytokine stimulation can lead to upregulation of NK1.1 and other NK cell-related markers in CD8^+^ T cells, perhaps suggesting that iNKT1 cells acquire NK1.1 expression in a similar manner (101). And finally, since iNKT1 cells are primarily the only cells expressing NK1.1, learning about iNKT cell tissue localization through the use of this marker is by necessity restricted to this subset. Despite these drawbacks, some aspects of the tissue distribution patterns of iNKT cell subsets could be gleaned from early studies.

Of the subsets, iNKT1 cells have been indirectly demonstrated to remain long-term thymic residents and accumulate over time. When congenically marked thymic lobes were transplanted in recipient mice, while different kinds of iNKT cells were observed early after transplantation, only NK1.1^+^ iNKT cells persisted in the thymus as time progressed (34). In fact, over 50% of the mature αβ TCR^+^ cells remaining of donor origin were these likely iNKT1 cells that were maintained for a long period of time. This finding is in stark contrast to the conventional T cell population that is rapidly turned over in the thymus (102, 103). One possible explanation for thymic retention of iNKT1 cells is that T-bet drives expression of the chemokine receptor CXCR3, allowing them to be maintained in the thymus due to high levels of the cognate CXCR3 ligand CXCL10 (35). Another explanation for this phenomenon could be that T-bet in iNKT1 cells induces the expression of the gene *Il2rb* coding for the protein CD122 (73), thereby supporting the response to the trans-presented IL-15 cytokine (104). This cytokine is produced by cells in the thymic medulla and not only serves as a survival cytokine for iNKT1 cells but also help stabilize T-bet itself in those cells (70, 105). In addition, IL-15 has been previously shown to upregulate CD69 in cells sensitive to this cytokine and indeed, iNKT1 cells do express high levels of CD69 (106, 107). Because ectopic overexpression of CD69 prevents thymic egress of conventional T cells (108), this IL-15-induced CD69 could potentially also play a role in iNKT1 cell thymic retention.

Despite the thymic retention, NK1.1^+^ iNKT cells are also found in the periphery. Interestingly, large numbers of NK1.1^+^ iNKT cells are found in the liver (109). This could be linked back to iNKT1 cells expressing T-bet and their sensitivity to IL-15. As previously mentioned, T-bet expressing cells also concomitantly express CXCR3 while IL-15 has been shown to condition cells to express CXCR6 in humans (110). The ligands for both these chemokine receptors (CXCL9/CXCL10 for CXCR3 and CXCL16 for CXCR6) are present in abundant quantities in the liver (111–113). Thus, by following their chemotactic gradients, it is not surprising that iNKT cells compose 20–30% of T-lymphocytes in the liver (30, 94). Moreover, liver iNKT cells also establish strong residency upon arrival, as evidenced by their reduced circulation in parabiotic mice (94). Long-term residency by these lymphocytes has been proposed to be due to the high expression of the transcription factor Hobit (114). Induced by both T-bet and IL-15, Hobit has been shown to be preferentially expressed in liver iNKT cells, preventing their egress from the liver (114), although a recent study disputes this finding (115). Nevertheless, thymic iNKT1 cells also express high levels of Hobit perhaps suggesting they might maintain their residency in a similar manner (116).

With B6 mice remaining a popular mouse model to study iNKT cells, iNKT2 and iNKT17 cell localization has been largely understudied. While in some cases, there has been some direct evidence of a specific subset, iNKT2/iNKT17 presence in peripheral tissues has instead been frequently inferred, either by chemokine/cytokine receptor expression or by their cytokine secretion profile. iNKT17 cells, in particular, were initially identified as IL-17 producing iNKT cells within the NK1.1^−^ population by several groups (117, 118). Thereafter, using RORγt-GFP reporter mice, a unique population of iNKT cells was identified in the thymus that was dependent on RORγt for secretion of IL-17 (119). Since RORγt expression is strongly correlated with expression of the chemokine receptor CCR6, iNKT17 cells are specifically directed to the skin (120, 121). Additionally, expression of this chemokine receptor also endows some iNKT17 cells to enter lymph nodes as they are enriched in peripheral lymph nodes compared to the other sub-lineages. Similar to CCR6, expression of CD103 is also high on iNKT17 cells, leading to preferential retention of these cells in the skin, where epithelial cells express the CD103 ligand, E-cadherin (121, 122). iNKT17 cells also uniformly express high levels of the protein Syndecan-1 (CD138), although the reason for why they express this is unknown (123).

Insight into iNKT2 cell localization, however, has been further hindered by the lack of unique markers defining this subset. Unlike iNKT1 and iNKT17 cells, cytokine secretion is insufficient to specifically identify iNKT2 cells since IL-4 is also secreted by iNKT1 cells. Additionally, while iNKT2 cells express high levels of GATA-3, iNKT1, and iNKT17 cells also express this transcription factor, albeit at slightly lower levels (19). And finally, although expression of the cytokine receptor IL-17RB (specific for the cytokine IL-25) on iNKT cells has been demonstrated to enrich for IL-4/IL-13-secreting cells, iNKT17 cells also express this receptor, thereby preventing the use of this marker to specifically distinguish iNKT2 cells in tissues (72).

Through the use of reporter mice and transcription factor staining, a recent study has resolved these ambiguities by shedding substantial light on iNKT cell tissue distribution as well as location within tissues (77). In this seminal study, iNKT cell subsets were identified by their transcription factor expression and analyzed in many different tissues. Additionally, by developing a technique called histocytometry, the authors were able to identify the intra-tissue localization of the iNKT cell subsets. For example, it can now be appreciated that approximately 70% of the thymic iNKT cells, irrespective of subset, reside in the medullary space. This could be due to greater accessibility to homeostatic/survival cytokines (IL-15 for iNKT1 and IL-25 for iNKT2/iNKT17) in the medulla as well as chemokine-mediated trafficking. Remarkably, the relative iNKT subset distribution within tissues is not equivalent across different strains of mice as evidenced by strain-specific iNKT cell subset distribution patterns (77). For example, skin-draining lymph nodes were largely enriched for iNKT17 cells in the NOD background and to a lesser extent in B6 mice. However, iNKT2 cells were the principal subset present in these lymph nodes in BALB/c mice. Other tissues also similarly contained different ratios of the subsets across the strains. It is currently unclear if this corresponds merely to the proportion of each subset generated in the thymus in different strains, since BALB/c mice generate significantly more iNKT2 cells, or if strain-specific tissue-homing biases also exist.

New subsets of iNKT cells besides the three described here have also been identified. iNKT cells producing IL-10 are abundant in adipose tissues, where they make up approximately 30% of all T cells (124). Acquiring the moniker iNKT10 due to their ability to produce IL-10, these cells express low levels of PLZF and are dependent on the transcription factor E4BP4 for their functional competence (42). Although these cells are thymically derived as they are absent in adipose tissues of athymic nude mice, they could not be identified in detectable numbers in the thymus of a WT mouse (42). However, in mice expressing a transgene with a modified TCRβ chain that results in fewer iNKT cells due to improper signaling, more iNKT10-like cells were observed in the thymus that preferentially homed to adipose tissue (125). Currently, though, how they arise in a WT mouse is unknown. Therefore, it is possible that one of the three thymic subsets gives rise to this new subset that differentiates in the periphery. What and how specific cells home and differentiate within adipose tissue is uncertain. It is conceivable that due to their expression of T-bet and ability to produce IFNγ after stimulation with PMA/ionomycin, they are cells that deviate from the iNKT1 lineage due to the adipose tissue microenvironment (42).

Another subset that has also received attention of late is the iNKT_FH_ subset, which, analogous to the conventional T_FH_ population, expresses Bcl6 and helps in antibody class-switching and somatic hypermutation (43, 45). This population was initially described in secondary lymphoid organs upon immunization with antigen in conjunction with αGC, prompting these cells to form stable contacts with B cells and induce germinal centers through the secretion of IL-21. Since this subset has been found in the spleen and iNKT1 cells are also found in higher numbers in the B cell zone (77), perhaps iNKT_FH_ cells represent another branch-off subset from the iNKT1 lineage. This would suggest that iNKT1 cells are somewhat plastic in the periphery and can adopt other fates based on the inflammatory cues they receive.

## iNKT Subset Functions at Steady State

Although T cells commonly circulate throughout the host body, so, they can properly survey all sites for any perturbations, many cells also establish long-term residency in various tissues (126). After a primary immune response has been cleared, a proportion of the antigen-specific cells are retained in the tissue to guarantee a faster response in the future. In the absence of any immune response, however, these cells are not quiescent and sessile but rather dynamically interact with other cells in the tissues to shape their microenvironment in crucial ways. Perhaps owing in part to their memory phenotype, iNKT cells similarly establish long-term residency in several different tissues (42, 94, 127). Beyond that, even without establishing residency, they play important roles in maintaining homeostasis even in steady-state conditions. For instance, their effector status enables them to readily secrete cytokines upon stimulation, which can have dramatic consequences for their surroundings (19, 77). Thus, they serve as a rheostat for how nearby cells acquire phenotypes that correspondingly influence tissue equilibrium.

Ample evidence exists that iNKT cells in the thymus skew the thymic microenvironment in substantial ways (Figure 1). Importantly, the ratio of the subsets affects the phenotypes of other conventional cells. For example, iNKT2 cells in the thymus affect the phenotype and functionality of CD8^+^ T cells. Usually, thymic CD8^+^ T cells exhibit naïve characteristics and display antigen-response kinetics that are delayed compared to memory CD8^+^ T cells. Through the use of IL-4 reporter mice, it was discovered that thymic iNKT2 cells constitutively produce IL-4 (19). This IL-4 conditions the surrounding CD8^+^ T cells to upregulate CXCR3 and Eomes and exhibit memory traits (128, 129). These “innate” memory CD8^+^ T cells display antigen-response kinetics reminiscent of memory cells despite never having encountered antigen previously (130). In so doing, they can play a major role in combating chronic viral infections by mounting rapid and robust responses (131, 132). Mutant mice with larger numbers of iNKT2 cells compared to wild-type mice or different strains of mice that endogenously produce large numbers of iNKT2 cells consequently have larger numbers of innate memory CD8^+^ T cells. For example, only ~15% of the total iNKT cells in 8-week-old B6 mice thymi are iNKT2 cells while similarly aged CBA and BALB/c mice have ~40 and ~50%, respectively (19). The IL-4 produced by these iNKT2 cells has been directly demonstrated to affect the numbers of innate memory CD8^+^ T cells, with B6 mice thymi possessing <4% while CBA and BALB/c mice thymi contain ~30 and ~60% innate memory CD8^+^ T cells, respectively.

In addition to affecting the CD8^+^ T cells, the IL-4 produced at steady state by iNKT2 cells also conditions SIRPα^+^ thymic dendritic cells (DCs) to upregulate and produce the chemokines CCL17 and CCL22 (19). These chemokines interact with CCR4, also expressed by iNKT2 cells, perhaps implying a positive feedback loop whereby iNKT2 cells are drawn to the medulla by these chemokines where they enact their effects and further ensure their continued presence due to their sustained production of IL-4. Regulatory T cells (T_regs_) appear to also be increased in number and proportion by the iNKT2-produced IL-4 (133). These T_regs_ exhibit more of an activated phenotype and, in fact, have a greater suppressive capacity in an immune response. Recent data have also identified IL-4 as an inhibitory cytokine for early thymic progenitors (ETPs) to commit to the T cell lineage (134). ETPs stimulated through the IL-4 receptor upregulated the myeloid-specific transcription factor C/EBPα, presumably halting their development into T cells. It would be curious to see if mice with a higher frequency of iNKT2 cells had correspondingly fewer ETPs seeding the T cell pool in the thymus. Finally, IL-4 promotes thymic egress of SP4 thymocytes in a S1P/S1PR1-independent manner (135). Although how IL-4 leads to an accumulation of SP thymocytes is currently unknown, it is clear that the pleiotropic effects of IL-4 by iNKT2 cells markedly change the thymic landscape, reinforcing their importance in tissue maintenance.

Significantly fewer MHC-II^hi^ Aire^+^ medullary thymic epithelial cells (mTECs) exist within the thymus of CD1d^−/−^ mice compared to the B6 control mice (105). Aire is a transcription factor exclusively expressed in mTECs that promotes the expression of peripheral tissue antigens and tolerance of developing SP thymocytes. Both central tolerance of SP4 thymocytes and generation of T_regs_ depends on MHC-II and Aire expression by mTECs. Reduction of the number of cells capable of carrying out these tasks compromises both of these functions (136). mTECs in a CD1d^−/−^ mouse are enriched for an immature phenotype (MHC-II^lo^ Aire^−^). Interestingly, this mTEC developmental arrest is critically dependent on RANKL expression by NK1.1^−^ cells iNKT cells, suggesting a potential other role for iNKT2 (and possibly iNKT17) cells in the thymus beyond their production of IL-4. It would be of further relevance to identify if BALB/c mice, which have much higher numbers of iNKT2 and iNKT17 cells in the thymus, have an even more profound defect in mTEC maturation in the absence of CD1d than was described in B6 mice.

It remains unclear why iNKT2 cells play such key roles in influencing different thymic compartments when iNKT1 cells have been identified as long-term thymic residents. What role(s), if any, iNKT1 and iNKT17 might have in maintaining thymic homeostasis is currently unknown.

Substantially less evidence exists for iNKT subsets impacting steady-state functions of other tissues. Production of IL-4 by iNKT2 cells continues to condition the peripheral tissues by contributing to the high IgE levels found in the sera of BALB/c mice as well as promoting a proportion of CD4^+^ T cells in the mesenteric lymph nodes (mLNs) to constitutively express the activated form of the transcription factor STAT6 (phospho-STAT6) (19, 77). Activated STAT6 translocates to the nucleus from the cytosol and promotes expression of GATA-3, implicating iNKT2 cells in potentially influencing the “T_H_2-bias” observed in BALB/c mice (137). Beyond this IL-4-mediated role of iNKT2 cells, our understanding of iNKT subset functions at steady-state in peripheral tissues is limited. Parabiosis experiments have determined that iNKT cells establish long-term residency in hosts in the liver and the lung (94, 127). In the liver, based on their expression of NK1.1, the resident cells are largely iNKT1 while expression of IL-17RB suggests an enrichment of iNKT2/iNKT17 cells in the lung. Although it is possible that the reason for their tissue residency is simply to act as sentinels that kickstart the overall immune response during an infection, tissue-resident lymphocytes quite frequently have roles beyond that. Thus, future experiments where iNKT cells are prevented from accumulating in those tissues, perhaps by conditional deletion of chemokine ligands in those tissues, should help illuminate how iNKT subsets are affecting tissues in non-infectious settings.

Recently, though, the increased attention paid to iNKT10 cells has uncovered some interesting functions of these cells in maintaining adipose tissue homeostasis. Experiments conducted using parabiotic mice demonstrated that iNKT10 cells establish long-term residency in adipose tissue where they support an immunosuppressive environment (42). Upon stimulation, over half of them secrete IL-10, which helps induce an anti-inflammatory “M2” macrophage phenotype. Moreover, in contrast to other peripheral iNKT cell subsets, these cells produce high amounts of IL-2 upon stimulation. This, in conjunction with the IL-10, also promotes T_reg_ expansion with a highly suppressive phenotype. This supports the idea that iNKT cells in the adipose tissue might also be producing these two cytokines at steady state, but this remains to be formally demonstrated. Although many iNKT10 functional features have primarily been uncovered by stimulating these cells with αGC, the cells also express high levels of PD-1 and *Nr4a1* even at steady state. This could indicate that the iNKT cells perhaps receive continuous TCR-mediated signals in the adipose tissue (42). Indeed, adipocytes themselves display high levels of CD1d molecules. Yet, the nature of the lipids that might be presented to iNKT10 cells by adipocytes remains to be discovered.

## iNKT Subsets in Immune Responses

Because of their varied responses, iNKT cells have been demonstrated to be involved in myriad immune responses in which they can be either protective or pathogenic (138, 139). In mice infected with *Streptococcus pneumoniae*, iNKT cells produce IFNγ within hours of infection (140, 141). Preventing iNKT cells from getting activated by using an antibody that blocks CD1d recognition by iNKT TCRs significantly increased bacterial loads, suggesting that iNKT cell activation contributed to bacterial clearance. Similar findings have been observed in other models in which mice have been infected with *Pseudomonas aeruginosa* or *Mycobacterium tuberculosis*, where iNKT cell deficiency also correlated strongly with increased bacterial burdens, hinting that iNKT cells are perhaps involved in helping clear different kinds of pathogenic bacteria (142–144). When mice deficient in iNKT cells were injected with fibrosarcoma cells, tumor progression was inhibited significantly only upon transfer of iNKT cells (145). Yet again, this protective effect was evident only when the recipient mice expressed CD1d, perhaps implying that the fibrosarcoma cells expressed lipids capable of activating iNKT cells when presented by CD1d. On the other hand, in a model of implanted osteosarcoma, 88% of CD1d^−/−^ mice rejected the tumors compared to only 24% of WT mice (146). The reasons for the contradicting roles of iNKT cells in tumor models remain unclear. Finally, transferring iNKT cells into the diabetes-prone NOD mouse conferred resistance to diabetes and, in one study, reduced the incidence of diabetes from 90 to 10% (147). In contrast, anti-CD1d treatment of (NZBxNZW)F1 mice led to increased protection from lupus (148, 149). Indeed, transferring iNKT cells from (NZBxNZW)F1 mice into healthy recipients was sufficient to transfer disease (150). Thus, iNKT cells can modulate the course of the immune response in a variety of manners, depending on the models being studied.

Although iNKT cell responses have been characterized in different diseased-state conditions, the specific iNKT cell subsets contributing to the response are largely unknown. Usually, the contribution of iNKT cells to an immune response is determined through the use of a CD1d^−/−^ mouse model and/or a TRAJ18^−/−^ mouse model, both of which lack iNKT cells. However, both of these mouse models have drawbacks. The original TRAJ18^−/−^ mouse was generated by introducing a neomycin resistance gene into the *Traj18* locus (151). Interestingly, these mice lack approximately 60% of their overall TCR repertoire due to an inability to express TCR rearrangements involving TRAJ genes upstream of *Traj18* (152), potentially due to the presence of the neomycin resistance gene (153). Thus, these animals not only lack iNKT cells but also a substantial proportion of their conventional TCR repertoire, potentially obfuscating some of the findings discovered in studies using these mice. Repeating these experiments in mice where *Traj18* was deleted without the presence of a neomycin resistance gene should help clarify the original results (154–156). In the case of the CD1d^−/−^ mouse model, new data have revealed that one of the four widely distributed knockout strains (157) continues to possess a small number of iNKT cells (158). Therefore, this specific knockout strain cannot be considered iNKT cell-deficient mice and conclusions obtained using these mice should be reassessed and instead be reevaluated using mice in which iNKT cells are completely absent (159, 160). Besides the use of these mouse models, the iNKT cell contribution to an immune response is further characterized only by the cytokines that affect the progression of the disease, frequently IFNγ and IL-4, but not the phenotype of the iNKT cells secreting those factors. Although this cytokine-secretion profile is more indicative of the specific subsets involved, it is often insufficient since iNKT1 cells are also capable of producing IL-4. Thus, a greater effort needs to be put forth to identify the subsets involved in any disease based on not just their cytokines produced but also by the transcription factors expressed.

A few studies have shed some light on the roles of specific iNKT cell subsets in diseases, albeit indirectly. One study used a transplantable tumor model to determine that CD4^+^ T cells negatively regulated tumor rejection. Upon further examination, it was discovered that CD4^+^ iNKT cells were the primary source of IL-13, creating an immunosuppressive environment that prevented tumor rejection (161). When these iNKT cells were depleted, either through the use of depleting antibodies targeting CD4 or CD1d^−/−^ mice, tumors were rejected at a significantly higher frequency. It is possible that the responding iNKT cells were iNKT2 cells due to their production of IL-13 and the fact that iNKT2 cells would have been abundant in the BALB/c mice in which these experiments were conducted (19, 72, 162). In addition, iNKT2 cells are enriched in the CD4^+^ population (19, 63), further lending credence to this idea, although it is perhaps worth revisiting these experiments using currently available tools.

Interestingly, there appears to be a tissue-specific bias associated with iNKT cells capable of mediating tumor rejection. When bulk iNKT cells from the liver were transferred into TRAJ18^−/−^ mice harboring a sarcoma, they were capable of halting tumor progression (163). However, bulk splenic and thymic iNKT cells were not similarly capable of rejecting the tumor growth. It was further determined that the DN hepatic iNKT cells were significantly better at tumor rejection compared to the CD4^+^ hepatic iNKT cells. This latter finding could perhaps be because upon stimulation, CD4^+^ iNKT cells tend to produce more T_H_2 cytokines that would be immunosuppressive compared to the T_H_1 cytokines that DN iNKT cells are more biased to produce (79). Indeed, when IL-4^−/−^ iNKT cells, irrespective of their tissue origin, were transferred into mice with tumors, they more potently rejected tumors compared to WT iNKT cells (163). Curiously, however, CD4^+^ and DN iNKT cells from different tissues all produced similar levels of IFNγ and IL-4, suggesting that although IL-4 does have an impact on tumor rejection, other differences between the subsets and their tissue-origin also likely affect the functions of the iNKT cells *in vivo*. The specific functional subsets associated with these findings, though, remain unknown because iNKT1 cells, for example, can be found in both the CD4^+^ and DN compartments (63). Thus, in light of what is known today, these experiments bear repeating with the use of transcription factor staining to identify any differences between the various sources of iNKT cells. It would be especially interesting to identify gene signature differences between the same iNKT subsets but from different tissues to understand if certain tissues impose functional variations.

In other studies, iNKT cells were also discovered to be relevant in airway hyperreactivity (AHR). One of the hallmarks of asthma, AHR features eosinophilia in the airways, enhanced mast cell growth, and increased levels of serum IgE (164). Conventional T_H_2 cells play a major role in exacerbating antigen-induced AHR. However, it appears that iNKT cells can prime the immune system initially to bias the response toward a T_H_2 phenotype. In fact, in several models of AHR, iNKT-deficient mice do not develop AHR (165, 166). Further, intratracheal administration of a lipid agonist and a protein antigen strongly activated the pulmonary iNKT cells to prime CD4^+^ cells specific for the protein antigen to polarize into T_H_2 cells (127). Indeed, IL-4/IL-13 produced by iNKT cells was discovered to be fundamental for mice to succumb to AHR in these studies, although this remains controversial since a later study found that iNKT cells were dispensable for airway inflammation (167). Despite this, the iNKT cells secreting the type 2 cytokines were subsequently identified as expressing IL-17RB, providing some evidence to suggest that the cells potentially promoting AHR were possibly iNKT2 cells (168, 169). Only IL-17RB^+^ cells, usually expressed by iNKT2 and iNKT17 cells (72), were capable of recapitulating the symptoms upon transfer into a iNKT cell-deficient mouse by secreting IL-4 and IL-13 (168, 169). Interestingly, in a different model of AHR in which mice were exposed to ozone instead of an allergen/antigen, IL-17 production by iNKT cells was also required in addition to IL-4/IL-13, perhaps implicating that iNKT17 cells also can contribute to AHR in certain contexts (170). The IL-17 produced by iNKT cells led to increased neutrophilia instead of eosinophilia in the airways and has been demonstrated in a separate study to be dependent on c-Maf, a transcription factor also involved in promoting the proper function of T_H_17 cells (171–173).

Administration of αGC intravenously in mice can activate the vascular-localized iNKT cells. In this fashion, the hepatic and the red-pulp splenic iNKT cells, which are primarily iNKT1 cells, respond within minutes by producing IFNγ and IL-4. Serum increases of both these cytokines can easily be detected in these conditions (77) and the IL-4 secreted under these conditions appears to have long-range effects as demonstrated by the increased phosphorylation of STAT6 in CD4^+^ T cells in other tissues, such as LNs, despite the iNKT cells in those tissues remaining unstimulated. Thus, blood-borne pathogens that are capable of activating iNKT cells could possibly activate iNKT1 cells due to their localization that could then condition T cells in distal tissues. Analogously, since iNKT2 cells are present in high numbers in the mLNs of certain mouse strains, oral administration of αGC largely activated these cells and caused them to secrete IL-4 in large quantities (77). However, perhaps due to a lack of proximity to the circulation, the IL-4 produced in this setting had primarily local effects, with only the CD4^+^ T cells in mLNs increasing their phospho-STAT6 levels while T cells in other tissues were unaffected.

Bacteria express their own lipids, some of which might serve as stimulatory antigens to iNKT cells. Viruses, however, hijack host machinery for their own purposes and thus, are devoid of any lipids themselves and thought to not activate iNKT cells directly. However, viral infections can lead to upregulation of CD1d by triggering toll-like receptors (TLRs) (174). Additionally, infection can also lead to activation of hypoxia-inducible factor, which in turn could alter the lipid metabolism and allow antigenic self-lipids to be presented to iNKT cells (175). Thus, viral infections could lead to activation of iNKT cells in a CD1d-dependent manner (176). Alternatively, activation of innate cells such as DCs through TLRs could prompt them to secrete pro-inflammatory cytokines such as IL-12 and IL-18 that consequently activate iNKT cells (177, 178). These activated iNKT cells can secrete IFNγ that promotes an antiviral response (179, 180). Thus, iNKT cells can participate in viral infections, potentially in a protective role. However, the specific subsets involved in viral clearance are unknown. Although the production of IFNγ by iNKT cells strongly suggests that the subset involved is the iNKT1 subset, this remains to be formally demonstrated.

Interestingly, a new study has highlighted that iNKT cells influence humoral immunity during Influenza A virus infection (181). A previous study had identified iNKT cells as important in curbing myeloid-derived suppressor cell (MDSCs) function in influenza infection (182). The MDSCs in influenza-infected mice suppressed influenza-specific immune responses, leading to high titers of the virus. In a CD1d-dependent manner, iNKT cells were able to restrict the activity of MDSCs and consequently boost the immune responses directed against influenza. Thus, a role for iNKT cells in combating influenza virus infection had already been established. In the recent study, the authors focused on how iNKT cells affect B cell responses in influenza infection. These cells influence B cell germinal center formation and antibody class switching despite not being iNKT_FH_ cells. The iNKT cells are the primary secretors of IL-4 early during the infection and express CXCR3, suggesting that they are possibly iNKT1 cells. CD1d-mediated interactions with CD169^+^ macrophages were critical for the production of IL-4 by the iNKT cells. This response underscores a novel role that iNKT1 cells potentially play in mounting an immune response against viral pathogens. Although why iNKT1 (if the cells are indeed iNKT1) cells are producing IL-4 and the iNKT2 cells are not is unknown. It could be due to a possible abundance of iNKT1 cells in the mediastinal LNs or it could be that the macrophages present lipids on CD1d only capable of activating iNKT1 cells. Confirming the specific subset involved in this immune response and why these cells are preferentially activated is paramount.

More recently, with iNKT10 cells entering the fold, a new role has been added to the growing list of iNKT cell roles (183). In a model of diet-induced obesity in which mice were fed with high-fat diets (HFD), iNKT cells were depleted from adipose tissues, although this was reversible once their diets were switched to standard fat diets (124). Mice lacking iNKT cells fed HFD weighed more, had large adipocytes, elevated fasting blood glucose levels, and increased insulin resistance. Furthermore, there was an increased infiltration of proinflammatory macrophages into the adipose tissues, an important intermediary step in the inflammation and pathogenesis associated with obesity. As mentioned previously, it is unknown what cytokines the adipose tissue-resident iNKT cells secrete at steady state to maintain healthy adipocytes. However, these iNKT10 cells are different from other tissue localized iNKT cells because of their expression of E4BP4 (42). When iNKT cells transfected with E4BP4 were stimulated, they secreted more IL-10 than E4BP4^−^ stimulated iNKT cells, directly correlating E4BP4 to IL-10 production in iNKT cells. In addition, upon stimulation, the adipose-resident iNKT cells were capable of expanding T_regs_ in the adipose tissue in an IL-2-dependent manner and the adipose T_reg_ population is substantially reduced in iNKT cell-deficient mice (42). Thus, the immunoregulatory role that the iNKT10 subset plays in adipose tissue to prevent obesity-related illnesses could be due to direct secretion of IL-10 (and possibly IL-2) at steady state.

## Human iNKT Cell Subsets

In humans, iNKT cell numbers are substantially more variable compared to the inbred mouse strains routinely used. Usually, their frequencies are lower in human blood compared to mouse blood (around 0.01–0.1% compared to 0.2–0.5% in mice), and their frequencies are more variable in other tissues when compared to the analogous mouse tissues (184, 185). Humans instead have larger proportions of other innate-like T lymphocyte populations, such as mucosal-associated invariant T cells and the group I CD1-restricted T cells (12). Despite the reduced frequencies, human iNKT cells can be isolated from healthy individuals and patients and analyzed for function and phenotype. Unfortunately, a rigorous manner of identifying human iNKT cells from clinical samples has not always been consistently employed. Many times, the cells were identified by staining for human NK-cell markers such as CD56 and CD161 (the human counterpart of NK1.1) but as in mice, these markers are also expressed on other T cell populations (186, 187). In other studies, iNKT cells were identified by using antibodies targeting the TCRβ chain used by these cells (TRBV25), but this is also problematic since other T cell populations also express this TCRβ chain (188). More recently, iNKT cells have been identified either through the use of αGC-loaded human CD1d tetramers or by using an antibody targeting the invariant TCRα rearrangement unique to iNKT cells (189, 190). Both tools have provided greater resolution into understanding iNKT cell function in humans.

Invariant natural killer T cells in humans can be broadly categorized as DN, CD4^+^, and a small percentage of CD8^+^ cells (185). There appears to be some functional conservation of these subpopulations between species since the DN cells tend to have a T_H_1 bias while the CD4^+^ cells have a T_H_2 bias, although the CD4^+^ cells are also capable of secreting T_H_1 cytokines (184, 191). This suggests that perhaps human iNKT1 cells are present in both the CD4^+^ and DN fractions while iNKT2 cells are primarily present within the CD4^+^ fraction. Further evidence to support this hypothesis stems from the fact that the DN cells express higher levels of several NK receptors compared to the CD4^+^ cells, similar to how murine iNKT1 cells primarily express the NK receptors (185, 191).

Interestingly, it has been shown that human iNKT cells also express high levels of PLZF compared to other T cell populations (37, 38). Additionally, the CD4^+^ iNKT cell population appears to express higher levels of PLZF as identified by mRNA levels, perhaps because more iNKT2 cells are present within this population. Indeed, iNKT cell numbers and phenotype appeared to be significantly altered when a patient with biallelic PLZF deficiency was analyzed (192). Human iNKT cells also require SLAM receptor-mediated signals for proper development because humans lacking the adapter SAP lack any observable iNKT cells (193). Despite these studies, whether or not functional iNKT cell subsets follow a similar developmental path in humans and mice has not been formally addressed.

The identification of functionally distinct human iNKT cell subsets with differential expression of master transcription factors, similar to what is observed in mice, is currently limited. Instead, the cells are usually sub-divided based on their cytokine-secretion profile and/or their expression of the CD4 coreceptor. For example, iNKT cells found in the cord blood of humans appear to have an intrinsic bias to secrete IL-17 and cannot produce IFNγ (194). Furthermore, a RORγt inhibitor selectively impaired IL-17 production by iNKT cells in different tissues, suggesting that some iNKT cells could indeed constitutively express RORγt (although this was not formally tested) that endows them with the ability to secrete IL-17 upon stimulation (195). Different iNKT subsets identified by their differential expression of CD4 could induce secretion of different isotypes of antibodies by B cells. In particular, CD4^+^ iNKT cells were unique in their ability to induce expansion of a CD1d^hi^ CD5^hi^ B_reg_ population (196). These cells, however, did not express CXCR5 or PD-1 at high levels when placed in co-culture with the B cells, suggesting that they are not likely to be iNKT_FH_ cells. As in mice, immunosuppressive functions were associated with CD4^+^ iNKT cells in various tumor settings, further implicating a functional distinction between CD4^+^ and CD4^−^ iNKT populations (197). In other models of autoimmunity, iNKT cell numbers were found to be reduced and functionally impaired in their ability to secrete IL-4, although whether this reflects the loss of a specific subset is unknown (138). Reduction in iNKT cell numbers was also observed in obese patients in the peripheral blood, and these numbers appeared to increase once the patients underwent bariatric surgery (124). Whether these cells are the human equivalents of the murine iNKT10 population remains to be explored. Thus, overall, there are primarily tidbits of information regarding functional diversity in human iNKT cells without a cohesive paradigm comparable to the one established in mice. Future work should focus on understanding iNKT cell function in diseased states with increased granularity, with special attention paid to linking function to transcription factors expressed by different cells.

## Concluding Remarks

Subset differentiation of iNKT cells is a complex and multifaceted process. Despite this complexity, the final subsets are surprisingly similar phenotypically to other cell types belonging to their corresponding functional group. For example, there is a striking similarity between iNKT1 cells, NK cells, T_H_1 cells, and ILC1 cells (198). Similarly, iNKT2 and iNKT17 cells share similarities to their γδ and ILC counterparts. What then makes iNKT cell subsets special when other cells occupy similar niches and respond similarly? Two different aspects provide iNKT cells with a unique ability to influence the immune response. First, their ability to recognize lipids in an antigen-specific manner allows these cells to sample an antigen space that would otherwise be unmonitored by conventional T cells. Second, the kinetics of their responses to antigenic stimulation allow the iNKT subsets to rapidly skew the course of the immune response in directed ways. By establishing an initial path for the immune response, iNKT cells have the potential to dictate how downstream adaptive cells are polarized and, consequently, how they respond. Thus, understanding the functional diversity within iNKT cells is essential to be able to manipulate the immune system. By gaining a greater understanding about iNKT cell subsets and their functions, one can hope to target specific subsets in an effort to influence various immune responses in the future.

## Author Contributions

Both authors wrote and edited the manuscript.

## Conflict of Interest Statement

The authors declare that the research was conducted in the absence of any commercial or financial relationships that could be construed as a potential conflict of interest. The reviewer SJ and handling Editor declared their shared affiliation.
